# A Compact Tunable Diode Laser Absorption Spectrometer to Monitor CO_2_ at 2.7 μm Wavelength in Hypersonic Flows

**DOI:** 10.3390/s100606081

**Published:** 2010-06-17

**Authors:** Raphäel Vallon, Jacques Soutadé, Jean-Luc Vérant, Jason Meyers, Sébastien Paris, Ajmal Mohamed

**Affiliations:** 1 The French Aerospace Lab, ONERA, BP72-29 avenue de la Division Leclerc, FR-92322 Chatillon cedex, France; E-Mails: rvallon@onera.fr (R.V.); jacques.soutade@onera.fr (J.S.); jean-luc.verant@onera.fr (J.-L.V.); 2 Von Karman Institute, 1640 Rhode-St-Génèse, Belgium; E-Mails: jmmeyers@uvm.edu (J.M.); paris@vki.ac.be (S.P.)

**Keywords:** gas sensor, tunable diode laser absorption spectroscopy, distributed feedback (DFB), laser velocimetry, CO_2_ monitoring, hypersonic flow, Mars atmosphere, 07.07.Df, 42.55.Px, 07.57.Ty, 42.62.Fi, 42.79.Qx, 47.40.Ki, 96.30.Gc

## Abstract

Since the beginning of the Mars planet exploration, the characterization of carbon dioxide hypersonic flows to simulate a spaceship’s Mars atmosphere entry conditions has been an important issue. We have developed a Tunable Diode Laser Absorption Spectrometer with a new room-temperature operating antimony-based distributed feedback laser (DFB) diode laser to characterize the velocity, the temperature and the density of such flows. This instrument has been tested during two measurement campaigns in a free piston tunnel cold hypersonic facility and in a high enthalpy arc jet wind tunnel. These tests also demonstrate the feasibility of mid-infrared fiber optics coupling of the spectrometer to a wind tunnel for integrated or local flow characterization with an optical probe placed in the flow.

## 1. Introduction

Mars, with its carbon dioxide atmosphere, will probably be the next planet visited by the human species. But before sending a man to Mars, we must master the entry phase of a space vehicle into that planet’s atmosphere. A high enthalpy hypersonic wind tunnel is one of the technical ways to reproduce on Earth physical conditions similar to those of a potential Martian atmospheric entry. However, such wind tunnels have been developed with air or nitrogen gas for modeling Earth reentry and now these facilities must be extended to run with carbon dioxide, which is a much more complex molecule. For more than a decade now, Tunable Diode Laser Absorption Spectroscopy (TDLAS) has been one of the most used optical tools to characterize the free stream parameters of hypersonic flows through measurements of probe species like NO, H_2_O, CO and CO_2_ [1,2]. However the setups are usually quite cumbersome because of the use of cryogenic lead salt diode lasers necessary to detect these molecules in the mid-infrared spectrum. We are developing a new absorption spectrometer based on a recently available antimony diode laser [3] for CO and CO_2_ probing. These diodes emit in the 2 to 3 μm spectral range where there are quite intense CO/CO_2_ lines for comfortable signal to noise ratio measurements under rarefied gas flow conditions. Such diodes have already been successfully used in combustion and shock tube studies [4–6]. Compared to the PbSe diodes used in our proceeding studies [2], they are non cryogenic as well as less complex (compared, for example, to a difference frequency generation scheme) allowing design of a quite miniature setup. To locally monitor carbon dioxide, the laser beam can be sent to a probe in the flow through infrared fibers. The concept of such a probe, comprising two arms equipped with mirrors and optical couplers and allowing only a small absorption path length in the flow, has already been laid down by Wehe *et al.* using near infrared laser diodes and fiber optics to probe H_2_O lines in high speed flows [7,8]. This configuration offers many advantages like avoiding unnecessary absorption outside the flow, making optical coupling with the wind tunnel’s test chamber easier and the probe could be positioned in the flow in the same manner as a classical Pitot probe.

**Figure 1.**
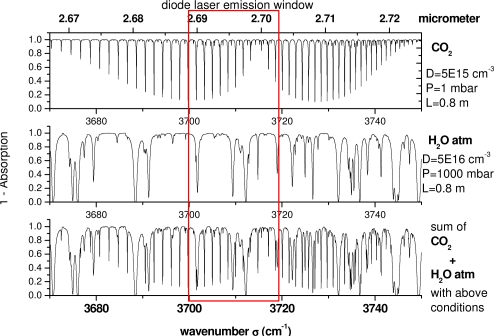
Diode laser emission window matched to low pressure CO_2_ (**ν1 + ν3**) and atmospheric H_2_O absorption lines in the 2.7 μm spectral region. The simulations are performed at values of density D, pressure P and absorption length L indicated in the figure.

The actual absorption spectrometer is equipped with a diode laser from Nanoplus emitting at 2.7 μm for CO_2_ probing and has been tested at the Von Karman Institute (VKI) LONGSHOT cold hypersonic wind tunnel and at the ONERA F4 high enthalpy wind tunnel. The diode emission window covers CO_2_ (ν1 + ν3) absorption lines of low and sufficiently high J, thus enabling the probing of a large range of temperatures and is therefore compatible with both wind tunnels. This is illustrated in Figure 1 where the diode emission window is matched to absorption line simulations under physical conditions typically encountered in these facilities using the HITRAN database [9]. The simulated gas has two components: CO_2_ at low pressure (about 100 Pa) mixed with some atmospheric pressure H_2_O usually absorbed outside the test section in the region of the spectrometer (but this is sometimes avoided through N_2_ purging). The simulations are performed for room temperature and for temperature of 800 K, which is typical for the F4 wind tunnel case.

**Figure 2.**
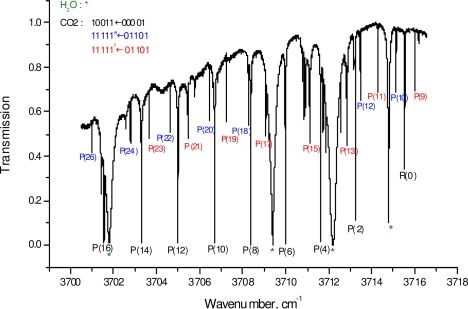
CO_2_ absorption lines (here for a 10 cm long cell filled with CO_2_ at a pressure of 4 mbar) present in the spectral emission window of the 2.7 μm diode.

Figure 2 presents the experimental total spectral emission spectrum (about 16 cm^−1^ wide) of the laser diode in our setup exhibiting the absorption lines of CO_2_ obtained when the laser beam crosses at room temperature a 10 cm long cell filled with CO_2_ at a pressure of 4 mbar. These lines are overlapped with atmospheric H_2_O lines absorbed outside the cell on a distance of about 1 meter.

The wavelength has been tuned only through temperature scanning of the diode (from −5 °C to 35 °C with the current fixed at 100 mA) and no correction of the emission intensity envelope has been applied in Figure 2. Such scanning takes a long time (200 s in our case). For our application of probing transient flows, a high speed scanning is required. This is possible through current tuning but this restricts the spectral window to about 1 cm^−1^ wide allowing the monitoring of only one or two absorption lines.

## 2. Results and Discussion

### 2.1. Optical Bench

**Figure 3.**
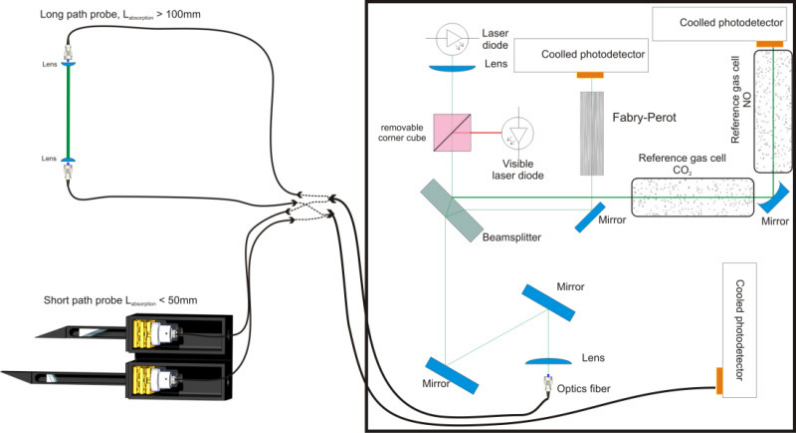
Optical bench setup and two methods of coupling to the flow.

The optical bench built is in the form of a three channel spectrometer where two channels are used for calibration: on the one hand, the beam travels through a low pressure gas reference cell for absolute wavelength calibration, and on the other hand, the beam travels through a Fabry-Perot interferometer having 4.9 × 10^−2^ cm^−1^ free spectral range (FSR) for relative wavelength calibration. These permanent and simultaneous calibration channels are necessary because one cannot rely on a beforehand calibration of the wavelength as there are many factors (harsh electromagnetic environment and long time delays before an effective flow run) which can cause wavelength drift in the diode set initially at certain desired conditions. The main part of the beam is coupled into an infrared fluorozirconate fiber (from IR photonics and having a core diameter of 85 μm) to bring the laser beam close to the flow. We can use either an optical probe for local measurement as tried by Wehe *et al.* [7] or two input/output fiber couplers for integrated path characterization as shown in Figure 3. The optical probe we designed allows obtaining an absorption path length of 4.24 cm in between its two arms (separated by a distance of 3 cm) with an angle of 45° with the respect to the flow axis. For one of the runs at the Longshot windtunnel, the use of short portion tubes allowed to reduce this path length to 3 cm.

All laser beams are detected by high sensitivity Peltier cooled photodetectors. The InAs type has been used for the calibration channels and the HgCdTe type, which has a better sensitivity and bandwidth, has been used for the channel dedicated to measure flow absorption. The three channel signals are simultaneously recorded by a 60 MHz bandwidth and 12-bits acquisition card. During a run, the laser wavelength is scanned on an interval of about 0.5 cm^−1^ containing the absorption lines to be monitored at a repetition rate ranging between 1 kHz and 10 KHz for a total duration of a few hundred of milliseconds.

### 2.2. Measurement Campaign at the Von Karman Institute (VKI) Longshot Wind Tunnel

The VKI Longshot free piston wind tunnel has been chosen for the first feasibility tests because of its non-chemically reacting nature of the flow where CO_2_ does not dissociate and is present at relatively high densities for a good signal to noise ratio. VKI has also a TDLAS system [10] running with a diode around 1.55 μm, but unfortunately it could not be operated simultaneously for comparison with our measurements. The VKI Longshot free piston tunnel is a short duration facility operating with nitrogen or carbon dioxide. It has been designed to generate very high Reynolds number hypersonic flows. Typical Reynolds numbers at Mach 15 range are from 5 × 10^6^ m^−1^ to 15 × 10^6^ m^−1^. It has a Mach 14 contoured nozzle of 0.43 m exit diameter and a 6 degree conical nozzle of 0.6 m exit diameter which can be used throughout the Mach number range from 15 to 20 using nitrogen and 10 to 15 using carbon dioxide.

A high precision incidence mechanism for pitch, roll, and yaw is mounted in the open-jet 4 m^3^ test section. This mechanism is used to maintain our optical probe in the correct position so that the laser beam has a 45 degree angle with the flow direction.

**Figure 4.**
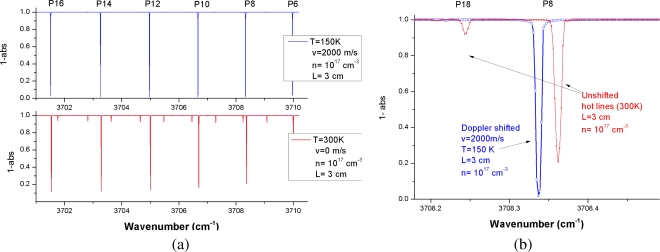
Simulated CO_2_ absorption spectrums for VKI run conditions.

During this measurement campaign, three runs at typical conditions of the wind tunnel were tested, two with CO_2_ flows and one with nitrogen seeded with 5% CO_2_, the latter aiming at probing Earth reentry flows. Figure 4 shows a simulated spectrum for typical hypersonic flow conditions at the Longshot facility (temperature of 150 K, velocity of 2,000 m·s^−1^, density of 10^17^ molecules·cm^−3^ and pressure of 100 Pa) for an absorption path length of 3 centimeters. In these conditions, the main absorption lines (of low quantum number J) to be used to characterize the cold free stream are nearly saturated whereas the “hot” (or high J) CO_2_ lines are hardly noticeable. One could think of increasing the absorption path length to bring a more comfortable signal to the hot lines but these lines cannot be exploited to characterize the free stream flow core as they will be disturbed by important absorption contributions from regions at higher temperatures than the core flow at 150 K.

For the Longshot experiments, the spectral window is chosen to monitor the P(8)e CO_2_ line for velocity measurements (Figure 4b) as the primary objective. It includes a hot line (P(18)f) which should not be visible in the experimental spectra for the low temperature section of the flow but can help to monitor hot portions of the flow. The experimental conditions for the three runs we tested are summarized in Table 1.

**Figure 5.**
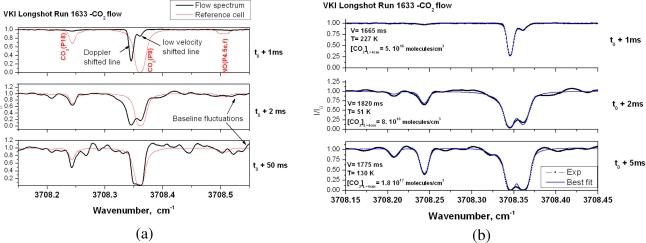
Wavelength and intensity calibrated spectra at different instances of the flow (a) and spectrum inversion (b).

Figure 5 presents typical spectra obtained during these runs. For all runs, the unshifted component of the P(8)e becomes more and more intense (Figure 5a) and this is interpreted as low velocity gas filling the probe, which is not vacuum tight. This gas component is at higher temperatures than in the free stream and explains also the appearance of the ‘hot’ P(18) line in the spectra of Figure 5. The spectra which are numerically fitted using a non-linear Levenberg-Marquart least-squares procedure are presented in Figure 5b. The data reduction uses the standard two-layer gas model described and justified in reference [2] where one layer corresponds to the free stream at high velocity and low temperature and the other layer accounts for all static gas at higher temperatures.

**Figure 6.**
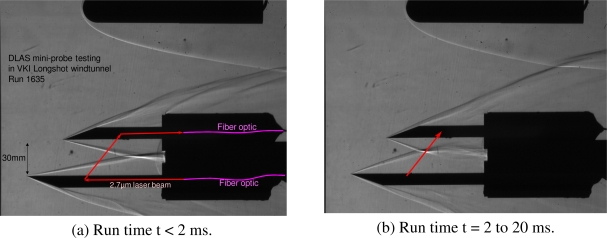
Schlieren images of the probe during VKI Run 1635 with optical beam path shown in red.

The high speed Schlieren video acquired during the run shows a small angle for shock layers at the beginning of the flow (Figure 6a). Unfortunately, after two milliseconds, these angles increase to bring the shock interactions on the path of the laser beam (Figure 6b). The large fluctuations of the shock-shock interaction zone induce disturbances of the laser beam and oscillations on the baseline of acquired spectra.

Even after these two milliseconds, the spectrum inversion on the CO_2_ P(8)e line yields free stream gas velocity, temperature and density with a relatively good agreement with results from free stream flow rebuilding based on measurements of plenum pressure and stagnation conditions in the free stream via a hemispheric probe [10,11].

**Figure 7.**
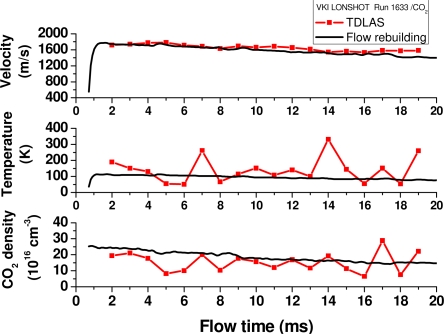
Compiled results for VKI LONGSHOT Run 1633.

Figure 7 shows a graphical presentation of this comparison. This close matching can be interpreted as that most of the shock layer region being probed has properties close to the free-stream. The interaction zone crossed is relatively small and its impact is more on bringing oscillations to the absorption spectrum baseline. This in turn impacts on the derived parameters like temperature and density whereas velocity is less affected as it depends less on line intensity profile. It is therefore difficult to infer accuracies to these parameters except for velocity for which the precision is estimated to be less than 10%.

Anyway, all derived values for velocity, temperature and CO_2_ density must be interpreted with care because of the laser beam crossing the shock interactions region in between the fingers of the optical probe. However, if we retain only the measurements for the first two milliseconds, this measurement campaign allows us to validate the feasibility of TDLAS probing at 2.7 μm in this wind tunnel as well as the use of infrared fluorine fiber optics coupled to a miniature probe with short absorption path in the flow.

### 2.3. Measurement Campaign at the ONERA F4 High Enthalpy Arc Jet Wind Tunnel

**Figure 8.**
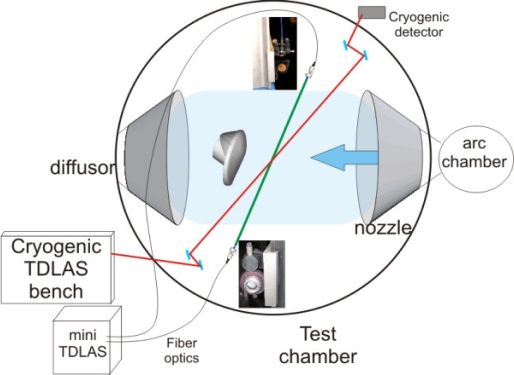
TDLAS setup experiment in the F4 wind tunnel

The ONERA F4 facility [12] is an arc jet wind tunnel that has been widely used for performing test campaigns for ESA Mars exploration projects such as Mars Sample Return Orbiter and Exomars. Such CO_2_ tests achieved high enthalpy level thanks to a 60 MW electrical rotating arc in a 10 L plenum chamber initially filled with gas at 10 to 40 bar at ambient temperature. The exact initial pressure, arc duration and power are the driving parameters to tune for the aimed flow conditions. During the arc generation, usually lasting from 40 to 120 milliseconds, the temperature and pressure will increase, up to 4,000 K and 400 bar, until a pyrotechnic valve is opened in the plenum chamber to release the test gas in a contoured nozzle generating a high enthalpy (up to 8 MJ/kg) hypersonic flow at velocities up to 3.5 km/s. Four contoured nozzles are available for F4, but only the nozzle#2 (4,490 expansion ratio) was used to perform CO_2_ test campaigns for flows at Mach numbers from 7 to 8 and unit Reynolds numbers close to 10^5^. The typical useful time for the flow is 200 milliseconds using carbon dioxide gas for the flow conditions we tested. Figure 8 presents the setup at the F4 facility of the mini-TDLAS system which has been run in parallel to a previous installed TDLAS device working with cryogenic lead-salt diode lasers for NO and CO probing [2].

The same fiber optics tested in the Longshot facility have been used to bring the laser beam of the 2.7 μm system into the test section The rest of the path across the flow has been orientated only with the help of the collimators at the fiber ends (Figure 8) since the optical probe could not be mounted together with the model into the test section. A more miniature probe is required and is currently being studied. The angle between the laser beam and the flow is 59.5 degrees for the 2.7 μm wavelength beam and the CO_2_ P(8) and P(10) lines have been chosen for this measurement campaign.

**Figure 9.**
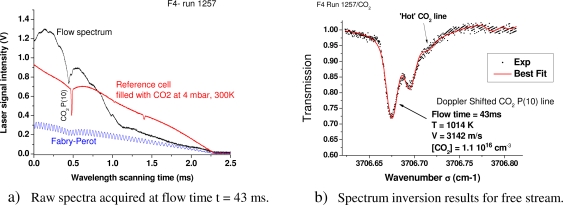
Example of absorption spectrum and derived measurements for the free stream of F4 run R1257 at flow time t = 43 ms.

The following measurements have been obtained during the ESA ExoMars tests campaign. An example of acquired spectra during the Run n° 1257 is shown in Figure 9a. The selected portion (and calibrated in wavelength and intensity) for spectrum inversion to derive the velocity, temperature and CO_2_ density flow is shown in Figure 9b.

**Figure 10.**
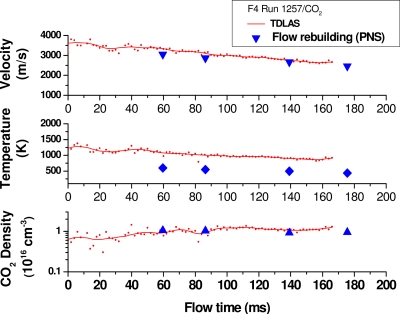
CO_2_ compiled results for F4 run 1257.

The velocity, temperature and density results derived from all acquired spectra (at a frequency of 2.5 kHz) for the F4 run n° R1257 are compiled in Figure 10. The velocity and temperature values decrease through time as the stagnation gas in the plenum is evacuated thereby decreasing the stagnation pressure and temperature conditions. This evolution of the stagnation conditions means also less dissociation for CO_2_ and therefore, an increase in CO_2_ density which is seen also by the DLAS results on density. Flow rebuilding for CO_2_ flows in this high enthalpy facility is presently difficult and time-consuming to perform because of the high levels of dissociation and non-equilibrium of the generated flows. However, the TDLAS results can be compared to a few results obtained with a parabolised Navier Stokes (PNS) code developed for air flows and presently being adapted to the CO_2_ case [13].

The CO_2_ flow is assumed to be in chemical non-equilibrium for the gas mixture created after arc heating process. The results for four flow rebuilding instants of run 1257 are tabulated in Table 2 and the velocity, temperature and CO_2_ density values are graphically reported in Figure 10: the TDLAS results match well with these points for velocity and CO_2_ density but present quite higher values for the flow temperature. The analysis of this discrepancy is still underway. On the measurement side, the possible causes are that the absorption line profile (for the CO_2_ P(8) case) is not well measured because of the low signal to noise ratio and also because of the disturbance caused by an overlapping weak line present at high temperatures. On the theoretical side, the PNS code still needs validation for CO_2_ flows and the TDLAS technique with an improved signal to noise ratio can be quite useful for this purpose.

## 3. Summary and Conclusions

We have developed a new transportable tunable diode laser spectrometer at 2.7 μm wavelength for hypersonic carbon dioxide flow characterization. This spectrometer has been tested during two measurements campaigns in two types of hypersonic wind tunnel: a free piston tunnel presenting reference cold conditions and an arc jet wind tunnel presenting dissociation-recombination chemistry. Both wind tunnels present harsh environments and low repetition occurrences for runs with short flow durations (200 ms at most). Nevertheless, we demonstrated on the few allocated runs the feasibility of this new compact TDLAS setup. We also demonstrated the feasibility of using a short absorption length optical probe coupled by infrared fiber optics for more local monitoring of a flow. We are presently improving this instrument through a new design for the probe so as to avoid having the laser beam crossing shock-shock interaction regions and through addition of another antimonide diode at 2.3 μm wavelength for simultaneous CO monitoring. The compact and portable instrument developed is also quite practical in other fields like environmental monitoring. We will publish soon the results of its application to monitor online trace gas species in the exhaust of airplane and automobile combustor engines.

## Figures and Tables

**Table 1.** VKI wind tunnel flow conditions and parameters used for TDLAS.

**Run number**	**Gas**	**Pressure, temperature**	**Absorption lines probes**	**Comment**
1633	CO_2_	230 Pa, 95 K at run time t = 10 ms	P(8)e and P(18)f for CO_2_	Schlieren not available, probe without protection tube
1634	CO_2_	230 Pa, 95 K at t = 10 ms	P(8)e and P(18)f for CO_2_	Probe with protection tube to reduce the absorption path length to 3 cm
1635	N_2_ with 5% CO_2_	130 Pa, 40 K at 10 ms	P(8)e and P(18)f for CO_2_	Probe without protection tube

**Table 2.** Numerical flow rebuilding values for four instants of F4 run 1257.

**Flow time (ms)**	**Stagnation conditions**		**Free stream conditions**								

	**Pressure Pi (bar)**	**Enthalpy H_i_ (MJ/kg)**	**Velocity (m/s)**	**Pressure (Pa)**	**Temperature (K)**	**MACH number**	**Density in cm^−3^**				
							**CO_2_**	**O_2_**	**CO**	**C**	**O**
60	180	6.5	3056	113	599	7.71	1.1E+16	1.1E+15	2.0E+15	1.4E+02	1.8E+13
86	149	5.5	2870	94	550	7.67	1.0E+16	7.5E+14	1.3E+15	1.2E+02	6.0E+12
139	110	4.5	2670	70	493	7.63	9.2E+15	4.2E+14	7.3E+14	1.0E+02	1.5E+12
176	93	3.5	2463	60	435	7.60	9.5E+15	1.8E+14	3.1E+14	1.0E+02	2.6E+11
